# Cluster analysis in 975 patients with current cough identifies a phenotype with several cough triggers, many background disorders, and low quality of life

**DOI:** 10.1186/s12931-020-01485-y

**Published:** 2020-08-20

**Authors:** Heikki O. Koskela, Tuomas A. Selander, Anne M. Lätti

**Affiliations:** 1 Unit for Medicine and Clinical Research, Pulmonary Division, Kuopio University Hospital, Postal address: PL 100, 70029 KYS Kuopio, Finland; 2 School of Medicine, University of Eastern Finland, Kuopio, Finland; 3 Science Service Center, Kuopio University Hospital, Kuopio, Finland

**Keywords:** Cough, Chronic cough, Asthma, Chronic rhinosinusitis, Gastroesophageal reflux disease, Cluster analysis

## Abstract

**Background:**

Recognition of disorder phenotypes may help to estimate prognosis and to guide the clinical management. Current cough management guidelines classify patients according to the duration of the cough episode. However, this classification is not based on phenotype analyses. The present study aimed to identify cough phenotypes by clustering.

**Methods:**

An email survey among employed, working-age subjects identified 975 patients with current cough. All filled in a comprehensive 80-item questionnaire including the Leicester Cough Questionnaire. Phenotypes were identified utilizing K-means partitional clustering. A subgroup filled in a follow-up questionnaire 12 months later to investigate the possible differences in the prognosis between the phenotypes.

**Results:**

Two clusters were found. The cluster A included 608 patients (62.4% of the population) and the cluster B 367 patients (37.6%). The three most important variables to separate the clusters were the number of the triggers of cough (mean 2.63 (SD 2.22) vs. 6.95 (2.30), respectively, *p* < 0.001), the number of the cough background disorders (chronic rhinosinusitis, current asthma, gastroesophageal reflux disease, 0.29 (0.50) vs. 1.28 (0.75), respectively, *p* < 0.001), and the Leicester Cough Questionnaire physical domain (5.33 (0.76) vs. 4.25 (0.84), respectively, *p* < 0.001). There were significant interrelationships between these three variables (each *p* < 0.001). Duration of the episode was not among the most important variables to separate the clusters. At 12 months, 27.0% of the patients of the cluster A and 46.1% of the patients of the cluster B suffered from cough that had continued without interruptions from the first survey (*p* < 0.001).

**Conclusions:**

Two cough phenotypes could be identified. Cluster A represents phenotype A, which includes the majority of patients and has a tendency to heal by itself. The authors propose that cluster B represents phenotype TBQ (Triggers, Background disorders, Quality of life impairment). Given the poor prognosis of this phenotype, it urges a prompt and comprehensive clinical evaluation regardless of the duration of the cough episode.

## Background

Cough is the most common symptom prompting people to consult a physician [1, 2]. Therefore, its management is of major importance. Current international cough management guidelines classify patients according to the duration of the cough episode: Acute (< 3 weeks), subacute (3–8 weeks), and chronic (> 8 weeks) [3–7]. However, this classification is not based on phenotype analyses.

Phenotypes are defined as the observable properties of disorders that are produced by the interactions of the genotype and the environment [8]. They represent groups with similar clinical characteristics, prognosis and/or therapeutic needs [9] and therefore, can be utilized in the clinical management of the disorders. In many common respiratory disorders, like asthma and chronic obstructive pulmonary disease, several distinct phenotypes have been documented [8, 10]. In chronic obstructive pulmonary disease, the international management guidelines nowadays lean on the documented phenotypes, highlighting their significance in the everyday patient management [11].

To the best of the authors’ knowledge, there are no previous investigations attempting to define cough phenotypes utilizing clustering, an acknowledged method to identify phenotypes [12]. Our recent, community-based survey identified 975 well-characterised patients with current cough [13, 14]. In the present study, we utilized clustering in this population to identify cough phenotypes.

## Methods

### Study design, setting, and population

This was a prospective, observational study conducted in 13,980 public service employees of two middle-sized towns in Finland (Jyväskylä, 8499 employees, mean 47 years with 80% females, and Kuopio, 5481 employees, mean 46 years with 78% females). In both towns, the employees represented a wide variation of professions, like nurses, teachers, caretakers, and sanitation workers. An invitation to the study and the first questionnaire were sent via e-mail to the employees’ e-mail addresses in March–April 2017. Answers were collected via an electronic reply form. One reminding message was sent if a subject had not responded within 2 weeks. The subjects reporting current cough in the first questionnaire formed the population for the cluster analysis.

In order to define the prognosis of the identified clusters, a second questionnaire was sent via e-mail in April 2018 to all participants who had suffered from cough during the first survey and who had provided a permission to follow-up. One reminding message was sent if a subject had not responded within 2 weeks. One phone contact was made if a subject had not answered within 2 weeks after the reminding message.

The study was approved by the Ethics Committee of Kuopio University Hospital (289/2015). Permission to conduct the study was obtained from officials of the towns. The invitation mail requesting participation in the study included detailed information about the study. The decision of the subject to reply was considered as an informed consent.

### Questionnaires

The first questionnaire included 80 items. There were questions about the subject’s household, pets, moisture damage both in their workplace and at home, family incomes, occupation, physical activity, smoking history, alcohol consumption, current medications, recent somatic symptoms, disorders diagnosed by a doctor, and general health-related questions. Many questions were adopted from two previous studies, the Health Behaviour and Health among the Finnish Adult Population study [15] and the Finnish National FINRISK study [16]. Asthma-, rhinosinusitis- and gastroesophageal reflux disease-related symptoms were enquired by questions currently suggested for epidemiologic studies [17–19]. Depressive symptoms were asked by utilizing the Patient Health Questionnaire-2 [20]. The patients who suffered from current cough also answered to detailed cough-related questions, like those about the frequency of coughing bouts and the duration of the cough episode. The latter question included seven alternatives: 1. Less than 1 week, 2. Longer than 1 week, but less than 3 weeks, 3. More than 3 weeks, but less than 2 months, 4. More than 2 months, but less than 1 year, 5. More than 1 year, but less than 5 years, 6. More than 5 years, but less than 10 years, 7. More than 10 years. They also filled in a list of potential triggers of cough as well as the Leicester Cough Questionnaire (LCQ), which was utilized to measure the cough-related quality of life (C-QOL) [21]. An English version of the first questionnaire can be found as a supplementary file (Additional file 1).

In the second questionnaire 12 months later the patients were inquired whether they suffered from cough and how long the cough had lasted. The questionnaire also included questions about current smoking, current moisture damage both at the workplace and at home, current pets, and current medications. Both questionnaires were first tested in a preliminary sample of 25 subjects and slightly revised before the final study.

### Definitions of variables that were formed on the basis of the raw data in the first questionnaire

Current asthma was defined as doctor’s diagnosis of asthma at any age and wheezing during the last 12 months [17]. Chronic rhinosinusitis was present if there was either nasal blockage or nasal discharge (anterior or posterior nasal drip) and either facial pain/pressure or reduction/loss of smell for more than 3 months [18]. Gastroesophageal reflux disease was present if there was heartburn and/or regurgitation on at least 1 day per week during the last 3 months [19]. The number of cough background disorders was calculated by summing up these disorders, giving a value from zero to three. Idiopathic cough was defined as absence of any of these disorders. Autoimmune disorder was defined as presence of a doctor’s diagnosis of hypothyreosis, rheumatoid arthritis, or other autoimmune disorders. Presence of depressive symptoms was defined as a Patient Health Questionnaire-2 score of three or more [20]. Symptom sum was calculated by summing all reported symptoms except those associated with airway disorders, giving a value from zero to 14. Trigger sum was calculated by summing all reported cough triggers. There were 11 triggers to be chosen. In addition, the LCQ question number 18 was utilized for speaking as a cough trigger, giving the maximal number of potential triggers 12. Allergy was defined as a self-reported allergy to pollens, animals or food. A family history of chronic cough was defined as the presence (now or in the past) of chronic (duration more than 8 weeks) cough in parents, sisters or brothers.

### Statistical analysis

All variables presented in the first questionnaire were included in partitional clustering with K-means method [12]. Dimension reduction and cluster analysis steps were performed using R statistical software version 3.5.1 (R Foundation for Statistical Computing, Vienna, Austria) with diffusionMap, NbClust and cluster packages.

At first phase, data were preprocessed. Right skewed (skewness> 1) variables were normalized with log(x + 1) function. Next, ordinal and continuous variables were scaled into 0–1 interval. Variable’s minimum value or the lowest class got value 0 and maximum value or the highest class 1. Binary variables remained unchanged. Value 0 indicated negative or ‘no’ alternative and value 1 positive or ‘yes’ alternative. After that, distance matrix between observations with scaled variables were calculated using Manhattan distance function. Diffusion maps dimension reduction method was applied to extract diffusion coordinates from distance matrix with function diffuse using default settings.

The number of clusters was evaluated by the 24 criteria provided by the software. After that, the extracted diffusion map coordinates were clustered into groups with k-means method. Cluster membership was added to original data for further analysis.

To validate the clustering, it was also performed by separating the population to two according to the hometown. Furthermore, the analyses were repeated by excluding those background variables with no plausible biological association with cough (like hometown, years of education, alcohol consumption etc.). The validation of the clustering also included the comparison of the prognosis between the clusters.

Statistical analysis between the clusters was performed by Mann-Whitney U test and chi-square test and the interrelationships of the variables was analyzed by the Spearman’s correlation coefficient (r_s_) using SPSS software version 22.0 (IBM SPSS Statistics for Windows. Armonk, NY, USA). Youden index (the value giving the best sum of sensitivity and the specificity) was utilized to define the cut-off values. The values are expressed by either means and standard deviations, medians and ranges, or percentages. A *p* value less than 0.05 was accepted as the level of statistical significance.

## Results

**Fig. 1:**
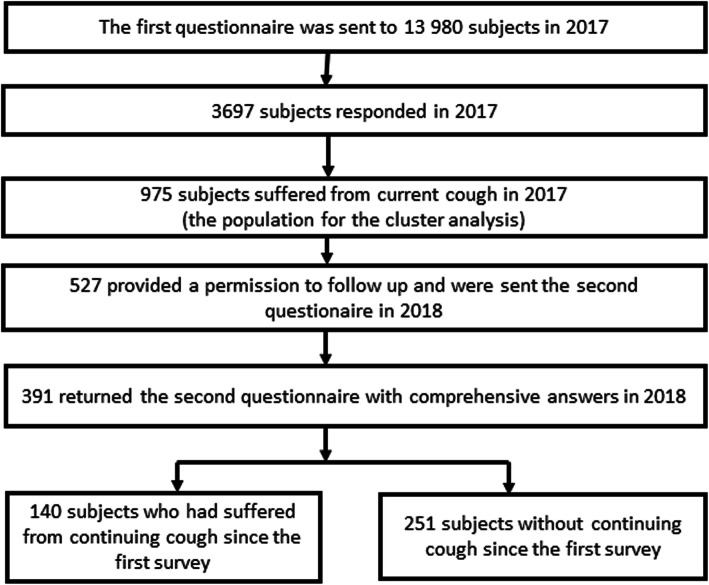
The flow chart

Of the 13,980 employees, 3697 (26.4%) responded. Their mean age was 47.8 (47.5–48.2) years and 82.6% were females. Of the 3697 responders, 975 suffered from current cough. They formed the population in which the clustering was applied (Fig. 1, Table 1). The proportion of missing values was less than 1% in all other questions except family income (2.5%) and acetylsalicylic acid intolerance (1.4%).

**Table 1:** The basic characteristics of the 975 subjects with current cough. Figures are means (standard deviations) or percentages unless stated otherwise

Characteristic	Data
Age, years	48.7 (10.6)
Female gender	83.8%
Body mass index, kg/m^2^	27.2 (5.2)
Current smokers	7.7%
Ever smokers	31.4%
Current asthma	19.3%
Chronic rhinitis	29.2%
Gastroesophageal reflux disease	17.4%
Chronic obstructive pulmonary disease	0.4%
Use of antihypertensive drugs	23.1%
Duration of the cough episode (median, range)	3–8 weeks (less than week – over 10 years)

**Fig. 2:**
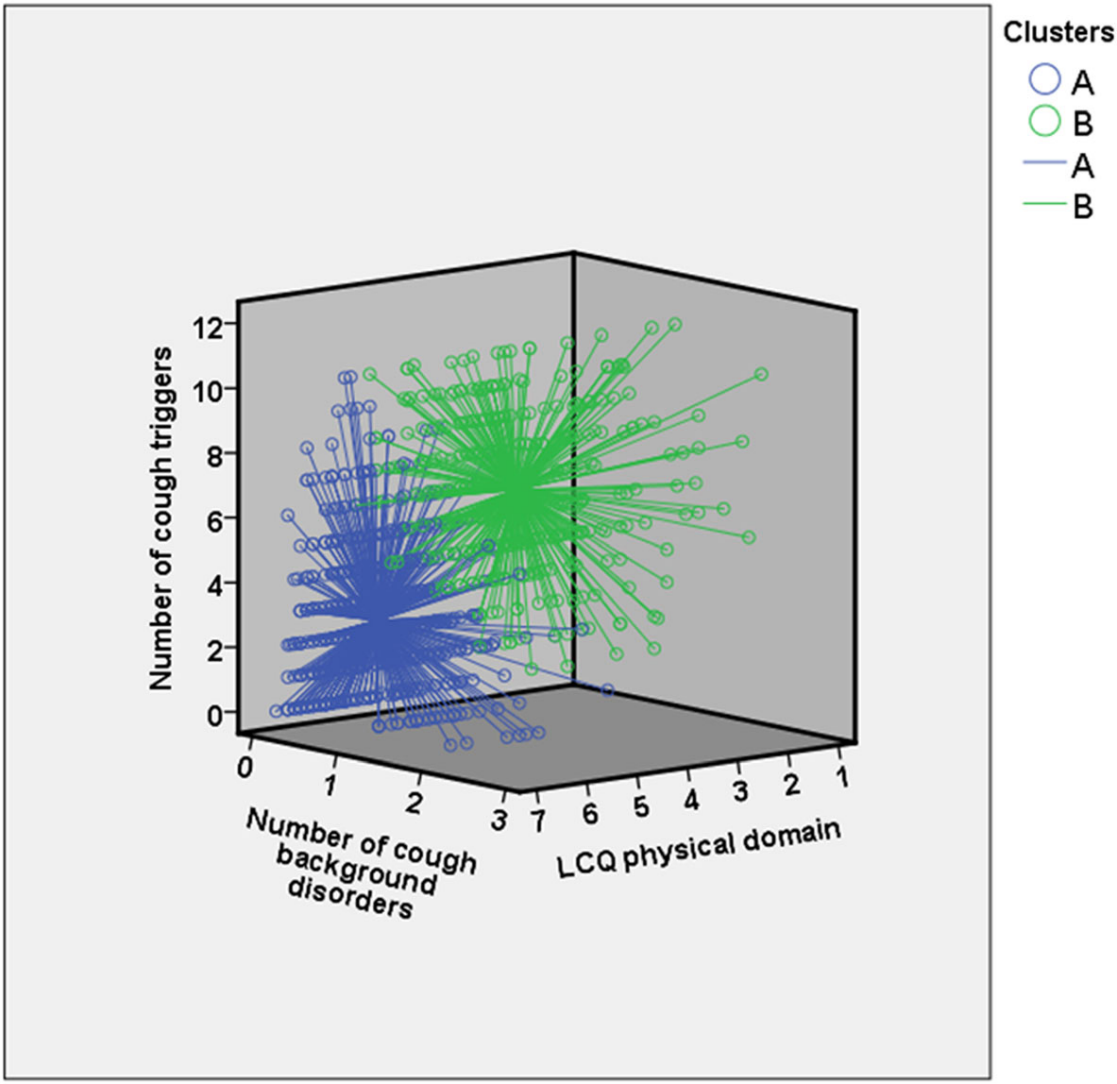
Each patient of the cluster A (*N* = 608, blue color) and cluster B (*N* = 367, green color) represented in a 3-dimensional figure according to the number of the cough triggers, the number of the cough background disorders, and the Leicester Cough Questionnaire (LCQ) physical domain. The marker of every patient is connected to the cluster mean value by a spike

Twelve of the criteria provided by the R statistical software suggested two clusters, six criteria suggested three clusters, and six criteria more than three clusters (Additional file 2). Therefore, the extracted diffusion map coordinates were clustered into two groups, called cluster A and cluster B. The cluster A included 62.4% of the population and the cluster B 37.6%. Table 2 presents those twelve variables that most strongly separated the clusters, according to the *p* value between the clusters, as well as 17 other variables of particular interest. The cluster B was especially characterized by several reported cough triggers, many cough background disorders, and low LCQ scores (Fig. 2). Of the various cough triggers, paints, fumes, poor indoor air quality, and strong scents most strongly separated the clusters. Of the three cough background disorders, asthma most strongly separated the clusters. Of the three LCQ domains, the physical domain most strongly separated the clusters. Table 3 presents the best cut-off values for the ten most important variables and their sensitivity and specificity values to identify the cluster B.

**Table 2:** The clusters and their defining variables among 975 subjects with current cough. The twelve most important variables and 17 variables of special interest expressed, in order of importance. The order was defined by the *p* value obtained by Mann-Whitney U test or chi-square test between the clusters. The values are expressed by either means (standard deviations) or percentages, unless stated otherwise

Order	Variable	Cluster A ***N*** = 608	Cluster B ***N*** = 367	***P*** value
1	Trigger sum	2.63 (2.22)	6.95 (2.30)	3.15 e-98
2	Number of cough background disorders	0.29 (0.50)	1.28 (0.75)	2.35 e-87
3	Idiopathic cough	73.8%	12.0%	3.27 e-77
4	LCQ physical domain	5.33 (0.76)	4.25 (0.84)	2.44 e-68
5	LCQ question 9 ^a^	6.06 (1.05)	4.34 (1.52)	5.45 e-66
6	LCQ total score	16.4 (2.48)	13.3 (2.75)	4.03 e-58
7	Dyspnea with wheezing	15.1%	65.4%	1.87 e-57
8	Strong paints or fumes as a cough trigger	18.8%	67.8%	8,12 e-53
9	Current asthma	4.3%	44.1%	3.38 e-52
10	Current medication for asthma	12.3%	57.5%	2.04 e-50
11	Poor indoor air quality as a cough trigger	39.3%	88.0%	7,67 e-50
12	Strong scents as a cough trigger	22.7%	70.8%	2,98 e-49
15	Chronic rhinitis	13.2%	55.9%	2,48 e-45
17	LCQ psychological domain	5.39 (0.97)	4.37 (1.06)	1.59 e-44
20	LCQ social domain	5.68 (1.02)	4.64 (1.12)	1.74 e-42
21	Number of doctor’s consultations due to cough during previous year	0.46 (1.03)	1.75 (2.16)	1.73 e-40
38	Chronic bronchitis	22.1%	58.7%	3.03 e-30
43	Current medication for allergies	24.0%	59.7%	1.59 e-28
62	Sick leave days due to cough during the previous year	1.31 (3.23)	4.39 (7.75)	1.34 e-19
67	Duration of the cough episode (median, (range))	3–8 weeks (< 1 week - > 10 years)	8–52 weeks (< 1 week - > 10 years)	1.32 e-14
70	Gastroesophageal reflux disease	10.9%	28.3%	5.84 e-12
72	Cough bout frequency (median, (range))	Once a day (less than once a week - several times a day)	Several times daily (less than once a week - several times a day)	1.49 e-11
76	Autoimmune disorders	9.5%	24.0%	1.65 e-09
83	Family history of chronic cough	40.5%	58.6%	7.02 e-08
94	Female gender	79.9%	90.4%	2.43 e-05
98	Body mass index, kg/m^2^	26.8 (5.15)	27.9 (5.24)	0.00012
120	Depressive symptoms	4.2%	7.9%	0.020
132	Age, years	48.1 (10.8)	49.5 (10.3)	0.074
140	Current daily smoking	8.7%	6.0%	0.16

^a^ Leicester Cough Questionnaire question number 9: “In the last 2 weeks, exposure to paints or fumes has made me cough” with a 7-step scale from 1 = all of the time to 7 = none of the time

**Table 3:** Sensitivity and specificity of the ten main variables to identify cluster B, utilizing the cut-off values giving the best sum of sensitivity and specificity (The Youden index)

Variable	Cut-off value	Sensitivity	Specificity
Trigger sum	≥ 5	85%	80%
Number of cough background disorders	≥ 1	88%	74%
Absence of idiopathic cough	Yes/no	88%	74%
LCQ physical domain	≤ 4.7	81%	71%
LCQ question 9 ^a^	≤ 5	73%	77%
LCQ total score	≤ 15.0	76%	76%
Dyspnea with wheezing	Yes/no	65%	85%
Strong paints or fumes as a cough trigger	Yes/no	68%	81%
Current asthma	Yes/no	56%	96%
Current medication for asthma	Yes/no	58%	88%

^a^ Leicester Cough Questionnaire question number 9: “In the last 2 weeks, exposure to paints or fumes has made me cough” with a 7-step scale from 1 = all of the time to 7 = none of the time

The duration of the cough episode was shorter in the cluster A than in the cluster B (Table 2) but the duration was not among the strongest variables to separate the clusters and there was a large overlap in the duration of the episode between the clusters. The best cut-off value for the duration to identify the cluster B was at least 3 weeks, which gave a sensitivity of 77% and specificity of 52%.

The validation analysis by separating the population to two according to the hometown gave similar results in the two populations: The suggested number of clusters was two in both towns and the main variables separating the groups were almost the same (Additional files 3 and 4). The analysis by excluding those background variables with no plausible biological association with cough did not change the main results, neither: The suggested number of clusters was two and the main variables separating the groups were the same (Additional file 5).

There were significant interrelationships between the most important variables: The number of cough triggers was associated with the number of cough background disorders and the LCQ physical domain (r_s_ = 0.44, *p* < 0.001, and r_s_ = − 0.44, *p* < 0.001), and the number of cough background disorders was associated with the LCQ physical domain (r_s_ = − 0.30, *p* < 0.001).

Of the 975 patients with current cough during the first survey, 527 provided a permission to follow-up and were sent the second questionnaire 12 months later (Fig. 1) and 391 (74.2%) returned it with comprehensive answers. Among them, there were 140 with continuing cough since the first questionnaire and 251 subjects without it. The cluster type significantly associated with the cough persistence since 27.0% of the patients of the cluster A and 46.1% of the patients of the cluster B reported of continuing cough (*p* < 0.001). The odds ratio of continuing cough at 12 months was 2.31 (1.52–3.53) for the cluster B.

## Discussion

In a population of 975 employed, working-age patients with current cough, two clusters could be identified. The cluster A was the larger of the two, including 62.4% of the population. The cluster B was smaller, including 37.6% of the population. The cluster B was especially characterized by several triggers of cough, many cough background disorders, and poor C-QOL. The prognosis of cough was clearly worse in the cluster B with almost half of the patients suffering from continuing cough up to 12 months from the first survey.

There are a wide variety of algorithms for clustering [12]. In the present analysis we applied partitional clustering with K-means method. An important step in clustering is to choose the variables to be included in the analysis. In order to avoid bias due to prejudice we included all information that was gathered by the first questionnaire. Since disorder-unrelated background variables can cause a bias to clustering we also performed the analysis by excluding the background variables which have no biologically plausible associations with cough. The main results did not change. Correctly identifying the number of clusters is another important step in clustering [12]. In the present analysis, we utilized the 24 criteria provided by the software and clearly the most frequently suggested number of clusters was two. To validate the clustering we performed the analysis by dividing the original population to two independent populations, according to the hometown. In both towns, two clusters was supported by most of the criteria and the main variables remained almost the same. However, the characteristics between these two subpopulations were rather similar and therefore, this kind of cluster analysis should be performed in other, different populations to further evaluate the validity of the present results.

The variable that most strongly separated the clusters was the number of the cough triggers. Ternesten-Hasséus et al. have previously shown that patients who report that environmental irritants trigger their cough are hypersensitive to the cough-provoking effect of capsaicin [22]. Therefore, we propose that the main pathophysiological feature separating the clusters A and B is the degree of the sensitivity of the cough reflex arc. Interestingly, paints, fumes, poor indoor air quality, and strong scents were the strongest triggers that separated the clusters. All these triggers can be labelled as chemical triggers, which are especially associated with the cough hypersensitivity to capsaicin [22]. The characteristics of the cluster B thus fit into the new concept of cough hypersensitivity syndrome, which indicates a long-standing hypersensitivity of vagal afferent nerves or an alteration of the central processing of their input regardless of the background disorder [23, 24]. Since the cluster B was associated with the prolongation of cough up to 12 months, the hypersensitivity of the cough reflex arc may have prognostic significance. This assumption is supported by the finding that an objectively measured cough hypersensitivity to hypertonic saline is associated with a poor 5-year prognosis in chronic cough [25]. The documented significant interrelationships between the number of triggers, number of background disorders and C-QOL suggest that they all may associate with the degree of the sensitivity of the cough reflex arc.

The second strongest variable that separated the clusters was the number of the cough background disorders, namely chronic rhinosinusitis, current asthma, and gastroesophageal reflux disease. In the cluster A, 73.8% of the patients had no cough background disorders. On the contrary, 88.0% of the patients of the cluster B had one or more cough background disorders. This is not an unexpected finding because all these disorders are well known to associate with chronic cough [3, 7]. It is well known that chronic obstructive pulmonary disease (COPD) also causes chronic cough. This disorder was also enquired in the first questionnaire but just 0.4% of the subjects reported it. The low prevalence of COPD in the present population may explain why cluster analysis did not raise it as one of the main defining variables. Furthermore, angiotensin-converting enzyme (ACE) inhibitors, which are commonly used for arterial hypertension, can also cause cough. As many as 23% of the subjects reported the usage of antihypertensive drugs but this was not recognized as a major cluster defining variable. Unfortunately, the questionnaire did not include a separate question for ACE inhibitors.

The third strongest variable was the level of impairment in the C-QOL. Low C-QOL can be regarded as the most important consequence of cough [21] and it is associated with repeated doctor’s consultations due to cough [26]. Therefore, it is not surprising that cluster B was also associated with frequent doctor’s consultations and many sick leave days due to cough.

Since this is, to the authors’ best knowledge, the first cluster analysis among patients with cough, we cannot compare our results with previous studies. Current international cough management guidelines classify patients according to the duration of the cough episode [3–7]. The classification by duration is based on the knowledge that certain cough background disorders are more common in prolonged cough than in shorter cough subtypes. However, the classification of cough by the duration of the episode is not based on phenotype analyses and thus, is more or less arbitrary. Furthermore, cough often has a relapsing and remitting course making the duration-based classification difficult to sustain [7]. In the present study, the duration of the cough episode was one of the many variables that could separate the clusters. However, it was far from the most important variables and there was a large overlap between the clusters. This finding speaks against episode duration -based classification of cough.

There are several shortcomings in the present study. First, the participation rate to the first survey was relatively low. It is possible that patients with severe cough have been more willing to participate than patients with mild cough. This may have led to an over-representation of phenotype B. In reality, the proportion of patients with cluster B-type cough is probably smaller than the 37.6% reported here. However, this bias probably did not affect the clustering analysis and its main results. Of note, the responders and non-responders did not differ with respect to age and sex distribution. Second, the population was rather homogenous, consisting of working-age, employed subjects. Old people and unemployed subjects are missing. Also, the number of current smokers was small. Third, the baseline questionnaire did not include the information about how many cough episodes they had had previously, i.e., the tendency of the cough to recur. Fourth, the analysis is based on questionnaire data only. Spirometry, laboratory, and x-ray data is missing. However, cough is an extremely common disorder [1, 2, 26] and primary care physicians usually do not have this information, neither, when deciding the management of cough.

The strengths of the present study include the large, community-based population. It is probably more representative than, for example, a population recruited from special cough clinics. The first questionnaire was comprehensive as the study was originally planned to investigate the risk factors, consequences and prognosis of cough. The cough background disorders were strictly defined as suggested in current literature. Validated questionnaires like PHQ-2 and LCQ were utilized. Furthermore, the prognostic data was provided.

## Conclusions

Two clusters could be identified in a large population with current cough. The authors propose that these clusters represent two cough phenotypes. Cluster A represents phenotype A, containing the majority of patients. This cough phenotype has a tendency to heal by itself. On the contrary, patients of the cluster B are at high risk of cough prolongation. They are especially characterized by several reported cough triggers, many cough background disorders, and severe impairment in the C-QOL. We propose an acronym TBQ (Triggers, Background disorders, Quality of life impairment) for this phenotype. The characteristics of the phenotype TBQ fit into the cough hypersensitivity syndrome [23, 24]. Duration of the cough episode was not among the most important variables to separate the phenotypes. Given the poor prognosis of the cough phenotype TBQ, it urges a prompt and comprehensive clinical evaluation regardless of the duration of the cough episode. In future, this kind of cluster analysis should be performed in other, different types of cough populations to further evaluate the validity of the presently identified phenotypes.

## Supplementary information

**Additional file 1.** The first questionnaire.

**Additional file 2.** The number of NbClust criteria suggesting the best number of clusters in the total population, among subjects living in Jyväskylä, and among subjects living in Kuopio. The maximal number is 24 criteria.

**Additional file 3. **The clusters and their defining variables among 520 subjects with current cough living in the town Jyväskylä. The ten most important variables are expressed, in order of importance. The order was defined by the *p* value obtained by Mann-Whitney U test or chi-square test between the clusters. The values are expressed by either means (standard deviations) or percentages, unless stated otherwise.

**Additional file 4. **The clusters and their defining variables among 444 subjects with current cough living in the town Kuopio. The ten most important variables are expressed, in order of importance. The order was defined by the *p* value obtained by Mann-Whitney U test or chi-square test between the clusters. The values are expressed by either means (standard deviations) or percentages, unless stated otherwise.

**Additional file 5. **The cluster analysis by excluding those background variables with no plausible biological association with cough among 975 subjects with current cough. The ten most important variables are expressed, in order of importance. The order was defined by the *p* value obtained by Mann-Whitney U test or chi-square test between the clusters. The values are expressed by either means (standard deviations) or percentages, unless stated otherwise stated.

## Data Availability

The datasets used and/or analysed during the current study are available from the corresponding author on reasonable request.

## Abbreviations

ACE: Angiotensin-converting enzyme
COPD: Chronic obstructive pulmonary disease
C-QOL: Cough-related quality of life
LCQ: Leicester Cough Questionnaire
TBQ: Triggers, Background disorders, Quality of life impairment
