# Supplementary material for: Cluster analysis in 975 patients with current cough identifies a phenotype with several cough triggers, many background disorders, and low quality of life
Source: Respir Res. 2020 Aug 20;21:219. doi: 10.1186/s12931-020-01485-y (PMC7441640; doi:10.1186/s12931-020-01485-y)
Supplement: Supplementary file 1 — Additional file 1. The first questionnaire. [file 12931_2020_1485_MOESM1_ESM.docx]

**cough in Savo and Central Finland**

**THE QUESTIONNAIRE**

**General questions**

**0. Your current employment**

1 City of Jyväskylä or some municipality-owned enterprise

2 City of Kuopio or some municipality-owned enterprise

**1. Gender**

1 male

0 female

**2. Year of birth___________**

**3. Marital status**

1 married/ in a registered relationship/ common-law marriage

2 unmarried

3 separated or divorced

4 widow/widower

5 can not define

**4. How many years of full-time education have you had?**

Please include both primary and secondary schooling

**______y**ears

**5. In addition to yourself, how many people live in your household?**

_____individuals

**6. At present, do you have any pets (also include farm animals if you live on a farm)?**

0 no (please proceed to question 8)

1 yes

**7. Do you have any of the following pets?**

no yes

Dog……………………………………………………….0…..1

Cat………………………………………………………. 0….1

Rodent (mouse, hamster, guinea pig etc.)……………......0…..1

Some other furry animal……………………….....……....0….1

Farm animals (cows, pigs etc.)…………………………...0….1

Some other animal…....…………………………………..0….1

**8. During the past 12 months has moisture damage been identified in your home?**

0 no

1 yes

**9. During the past 12 months has moisture damage been identified in your place of employment?**

0 no

1 yes

2 I have not been in employment for the past 12 months

**10. How large is your household’s yearly income (before tax and other deductions) adding the income of all of its members together?**

1 less than 15 000 e

2 15 000- 40 000 e

3 40 000 – 70 000 e

4. 70 000 – 120 000 e

5. More than 120 000 e

**11. Is your employment at present**

1 full-time

2 part-time

3 I am not working

**12. If you are working outside your home, what is your present or your most recent employment status?**

1 employer, entrepreneur

2 farmer, farmer’s wife

3 senior white collar worker

4 lower white collar worker

5 skilled blue collar worker (vocational college degree)

6 unskilled blue collar worker

7 I have not been employed outside the home

**13. During the past year (12 months) have you been unemployed or laid-off?** (please count the total time when not working i.e. current duration of unemployment or being laid-off)

1 not at all

2 0–1 month

3 2–3 months

4 4–6 months

5 7–11 months

6 12 months (i.e. for the whole year)

**YOUR USE OF HEALTH SERVICES AND YOUR HEALTH CONDITION**

**14. How many times in the past year (12 months) have you visited a doctor for any symptom whatsoever?**

If you have not visited a doctor at all in the past year, please write 0

_________times

.

**15. During the past year (12 months), how many full days have you been off work or unable to do your normal tasks because you have been ill?**

If you do not remember the exact number of days, please make an approximation. Do not include pregnancy in your count. If you have not been off work at all, please write 0

_______days

**16. During the past year (12 months) have you had any of the following illnesses either diagnosed or treated by a doctor?**

yes

asthma ................................................................................................. 1

chronic rhinitis………………………………..................................... 1

allergy (foods, pollen or to animals)…………….................................1

chronic obstructive pulmonary disease (= COPD)............................... 1

gastric distress (gastritis, gastric ulcer).…………………………....... 1

esophageal reflux disease (heartburn, gastroesophageal reflux )……..1

sleep apnea………………………………………………………….…1

depression ..............................................................................................1

other mental health problems ................................................................1

rheumatoid arthritis……. ......................................................................1

other connective tissue disorders (Sjögren’s syndrome etc.)……….....1

hypothyreosis……...…………………………………………………..1

arterial hypertension ..............................................................................1

elevated blood cholesterol .....................................................................1

diabetes...................................................................................................1

myocardial infarction…………….........................................................1

coronary artery disease, angina pectoris ……………………….......... 1

cancer ....................................................................................................1

spinal disc problems, other back disease……………………………...1

**17. Have you had any of the following symptoms or illnesses in the past month?**

yes

chest pain on exertion .........................................................................1

aching joints .......................................................................................1

back problems, back pain ...................................................................1

toothache ............................................................................................1

swollen feet ........................................................................................1

varicose veins .....................................................................................1

eczema ................................................................................................1

headache .............................................................................................1

insomnia .............................................................................................1

depressive symptoms...........................................................................1

other mental health problems .............................................................1

constipation ........................................................................................1

other gut problems (flatulence, diarrhoea) .......................................1

sciatica (back pain that radiates to the leg) ......................................1

**18. Have you used any of the following drugs in the past month?**

yes

cough medicines .............................................................................1

drugs for asthma…………………………………………………..1

drugs for rhinitis………………………..........................................1

drugs to treat allergies (for example, antihistamines).……………1

antacids (drugs to treat heartburn)………………………………..1

antihypertensives (drugs to treat high blood pressure)...................1

cholesterol lowering drugs..............................................................1

thyroid hormone (Thyroxine)……………………………………..1

insulin .............................................................................................1

drugs to treat diabetes ....................................................................1

analgesics (pain medicines) ...........................................................1

contraceptives................................ ………………………………1

tranquilizers ........................ ……………………………………..1

sedatives (sleeping pills) ................................................................1

antidepressants ...............................................................................1

vitamins or mineral supplements ...................................................1

hormone drugs to treat menopausal

or post-menopausal symptoms (for women) ..................................1

potency medicines (for men) .........................................................1

**19. Are you usually able to do the following physical actions?**

no yes

walking for about half a kilometer without stopping to rest ........................0....... 1

running for a short distance (about 100 meters)...........................................0....... 1

running for a longer distance (more than half a kilometer)..........................0……1

**20. At present, do you feel that your state of health is in general:**

1 good

2 quite good

3 about average

4 quite bad

5 bad

**21. How tall are you?**

_______ cm

**22. How much do you weigh? (wearing light clothing)**

_______ kg

**23. How often do you undertake either leisure-time physical activity or the equivalent while commuting to work that lasts for at least half an hour and which makes you at least slightly out of breath or sweaty?**

1 every day

2 4–6 times a week

3 3 times a week

4 2 times a week

5 once a week

6 2–3 times a month

7 a few times in a year or even less

8 I have a handicap or illness that makes it impossible for me to exercise

**24. How physically demanding is your normal work?**

In view of your present situation, choose which of the alternatives below is most appropriate for you

1 most of the time at work I am sitting and I do not walk very much

2 my job involves quite a lot of walking but I do not need to lift or carry heavy objects

3 my job involves a lot of walking and carrying or walking up stairs or hills

4 my job involves heavy manual labouring, I need to lift or carry heavy objects; my work involves digging, shovelling, hammering etc.

**25. How would you rate your present physical condition?**

1 very good

2 quite good

3 satisfactory

4 quite bad

5 very bad

6 I do not know

**SMOKING HABITS**

**26. Have you ever smoked at any time in your life?**

0 no (proceed directly to question 32)

1 yes

**27. During your life have you smoked at least 100 times?**

(cigarettes, cigars or pipes)

0 no

1 yes

**28. Have you ever smoked on a daily basis for at least one year? For how many years altogether?**

0 I have never smoked on a daily basis

1 I have smoked on a daily basis for ____ years

**29. Are you currently a smoker?**

(cigarettes, cigars or pipes)

1 yes, on a daily basis

2 occasionally

3 I am no longer a smoker

**30. When did you last smoke?**

If you are a regular smoker, please mark 1

1 yesterday or today

2 between 2 days and a month ago

3 between one and six months ago

4 between six months and a year ago

5 between one to five years ago

6 between five to ten years ago

7 more than 10 years ago

**31. On average, how much do you smoke now on a daily basis or did you smoke before you quit smoking?**

Please answer every point. Put 0, if you have never smoked that type of tobacco product at all

About ______ manufactured cigarettes every day

About self-rolled cigarettes every day

About______ pipefuls every day

About______ cigars every day

**32. Is there anyone who currently smokes either inside your home or in your workplace?**

0 no

1 yes

**ALCOHOL CONSUMPTION**

**33. In the past year (12 months) have you ever drank any alcoholic beverages (e.g. beer, wine, cider or strong alcoholic drinks)?**

0 I have not consumed any (please proceed to question 35)

1 yes

**34. How many glasses (counted as usual restaurant-sized glasses) or bottles of the following alcoholic beverages have you drank in the past week: if you have not drank any of the particular type of beverage, please mark 0 in that line**

About ____bottles (1/3 l) of medium strength or strong beer

About ____bottles (1/3 l) of alcopops /long drinks

About ____glasses of strong alcohol (restaurant sized glasses)

About ____glasses of wine or alcohol of similar strength (alcohol content over 5%)

About ____glasses of cider or low-strength wine (alcohol content about 5%)

**SYMPTOMS OF DISEASES OR ILLNESSES**

**35. In the past year (12 months), have you experienced wheezing or a whistling sound when you breathe**

0 no (please proceed to question 39)

1 yes

**36. Have you experienced a wheezing or whistling sound when you breathe at times other than when you are suffering from a flu or an upper respiratory tract infection?**

0 no

1 yes

**37. Have you experienced a shortness of breath at the same time when your breathing is wheezy or whistling?**

0 no

1 yes

**38. Has the wheezing or whistling sound when you are breathing disturbed your sleep in the past year (12 months)?**

0 no

1 yes

**39. In the past year (12 months) have you ever woken up due to an attack of a shortness of breath?**

0 no

1 yes

**40. In the past year (12 months) have you ever woken up due to a coughing fit?**

0 no

1 yes

**41. In the past year (12 months) have you experienced any of the following rhinitis-related symptoms?**

You can mark ”yes” to more than one option

no yes

nasal discharge (anterior or posterior nasal drip)…………………………..0………1

nasal blockage…………………………………….......................................0 .......... 1

facial pain or pressure………………...........................................................0 .......... 1

reduction/loss of smell …………………………………………………….0………1

**42. In the past year (12 months) have you experienced any of the following rhinitis-related symptoms in a prolonged fashion, for at least three months?**

You can mark ”yes” to more than one option

no yes

nasal discharge (anterior or posterior nasal drip)…………………………..0………1

nasal blockage…………………………………….......................................0 .......... 1

facial pain or pressure………………...........................................................0 .......... 1

reduction/loss of smell …………………………………………………….0………1

**43. Are you supersensitive to pain killing drugs (causing skin rash, swelling of your face, feelings of shortness of breath)?**

0 no

1 yes

**44. In the past 12 months have you suffered from heartburn and/or regurgitation?**

0 no (please proceed to question 47)

1 yes

**45. In the past 3 months have you suffered from heartburn and/or regurgitation?**

0 no (please proceed to question 47)

1 yes

**46. How often in the past 3 months have you suffered from heartburn and/or regurgitation?**

1 less often than once a month

2 at least once a month

3 at least once a week

4 every day

**YOUR MOOD**

**How often in the past two weeks have you been troubled by the following problems?**

**47. Little interest or pleasure in doing things?**

0 not at all

1 several days

2 more than half the days

3 nearly every day

**48. Feeling down, depressed, or hopeless?**

0 not at all

1 several days

2 more than half the days

3 nearly every day

**QUESTIONS CONCENTRATING ON COUGH**

**49. Do you have any close family members (father, mother, sisters, brothers) who are now suffering or have suffered from prolonged cough which has lasted over two months? (include also family members who have already passed away)**

0 no

1 yes

**50. Have you suffered from a phlegmy cough on most days or nights for at least three months yearly?**

0 no

1 yes

**51. During the past 12 months have you had a cough?**

choose only one option

0 not at all (please proceed to the last page)

1 yes, to some extent

2 yes, there have been periods at least two months long during which I have had a cough almost every day or even on a daily basis

**52. Do you think that there is some outside trigger or stimulus which makes you cough or worsens an existing cough?**

0 no (please proceed to question 54)

1 yes

**53. Which of the following triggers make you cough or worsens an existing cough?**

You can select more than one option

yes

Upper respiratory tract infection (”’flu”)…………………………1

Subfreezing air…………………………………………………....1

Physical exercise………………………………………………….1

Automobile exhaust fumes………………………………………..1

Poor indoor air quality…………………………………………….1

Proximity to animals………………………………………………1

Pollens……………………………………………………………..1

Cigarette smoke……………………………………………...…….1

Strong scents (perfumes, deodorants etc.)…………………………1

Strong paints or fumes………………………………………….…1

Something else………………………………………………...…..1

**54. In the past 12 months have you used cough medicines?**

(Both those purchased from a pharmacy and herbal remedies)

0 no

1 yes

**55. How many times in the past year (12 months) have you visited the doctor because of your cough?**

If you have not made any visits, then please mark 0

_______times

**56. How many complete days in the past 12 months has your cough forced you to be off work or be unable to do some normal tasks?** If you cannot remember exactly, an estimate will suffice. If you have not been off work due to cough at all, please put 0 in the line.

_______ days

**57. In the past two weeks have you had a cough?**

0 no

1 yes

**The last part of this questionnaire is intended only for those people who have had a cough in the past two weeks i.e. those people who responded “yes” to question 57.** Others can proceed to the last page of the questionnaire.

**THE FOLLOWING QUESTIONS ARE ONLY TO BE ANSWERED BY THOSE WHO HAVE BEEN SUFFERING FROM COUGH IN THE PAST TWO WEEKS**

**58. How often has your current cough been bothering you?**

1. Several times a day

2. Every day at least once a day

3. Four to six days in a week

4. Two or three times a week

5. At least once every week

6. Less than weekly

**59. For how long have you been troubled by your current cough?**

1. Less than one week

2. Longer than one week, but less than three weeks

3. More than three weeks, but less than two months

4. More than two months, but less than one year

5. More than one year, but less than five years

6. More than five years, but less than ten years

7. More than ten years

**60. Have you considered being examined by a doctor because of your current cough?**

0 no

1 yes

**61. How many times have you visited a doctor because of your current cough?**

Answer 0, if you have not visited a doctor due to your cough

_____times

The next questions are designed to assess the impact of cough on various aspects of your life. Read each question carefully and answer by choosing the response that best applies to you. Please answer all

questions, as honestly as you can.

**62. In the last 2 weeks, have you had chest or stomach pains as a result of your cough?**

1. All of the time

2. Most of the time

3. A good bit of the time

4. Some of the time

5. A little of the time

6. Hardly any of the time

7. None of the time

**63. In the last 2 weeks, have you been bothered by sputum (phlegm) production when you cough?**

1. Every time

2. Most times

3. Several times

4. Some times

5. Occasionally

6. Rarely

7. Never

**64. During the past two weeks, have you been tired because of your cough?**

1. All of the time

2. Most of the time

3. A good bit of the time

4. Some of the time

5. A little of the time

6. Hardly any of the time

7. None of the time

**65. In the last 2 weeks, have you felt in control of your cough?**

1. None of the time

2. Hardly any of the time

3. A little of the time

4. Some of the time

5. A good bit of the time

6. Most of the time

7. All of the time

**66. How often during the last 2 weeks have you felt embarrassed by your coughing?**

1. All of the time

2. Most of the time

3. A good bit of the time

4. Some of the time

5. A little of the time

6. Hardly any of the time

7. None of the time

**67. In the last 2 weeks, my cough has made me feel anxious.**

1. All of the time

2. Most of the time

3. A good bit of the time

4. Some of the time

5. A little of the time

6. Hardly any of the time

7. None of the time

**68. In the last 2 weeks, my cough has interfered with my job, or other daily tasks.**

1. All of the time

2. Most of the time

3. A good bit of the time

4. Some of the time

5. A little of the time

6. Hardly any of the time

7. None of the time

**69. In the last 2 weeks, I felt that my cough interfered with the overall enjoyment of my life.**

1. All of the time

2. Most of the time

3. A good bit of the time

4. Some of the time

5. A little of the time

6. Hardly any of the time

7. None of the time

**70. In the last 2 weeks, exposure to paints or fumes has made me cough.**

1. All of the time

2. Most of the time

3. A good bit of the time

4. Some of the time

5. A little of the time

6. Hardly any of the time

7. None of the time

**71. In the last 2 weeks, has your cough disturbed your sleep?**

1. All of the time

2. Most of the time

3. A good bit of the time

4. Some of the time

5. A little of the time

6. Hardly any of the time

7. None of the time

**72. In the last 2 weeks, how many times a day have you had coughing bouts?**

1. All the time (continuously)

2. Most times of during the day

3. Several times during the day

4. Some times during the day

5. Occasionally through the day

6. Rarely

7. None

**73. In the last 2 weeks, my cough has made me feel frustrated.**

1. All of the time

2. Most of the time

3. A good bit of the time

4. Some of the time

5. A little of the time

6. Hardly any of the time

7. None of the time

**74. In the last 2 weeks, my cough has made me feel fed up.**

1. All of the time

2. Most of the time

3. A good bit of the time

4. Some of the time

5. A little of the time

6. Hardly any of the time

7. None of the time

**75. In the last 2 weeks, have you suffered from a hoarse voice as a result of your cough?**

1. All of the time

2. Most of the time

3. A good bit of the time

4. Some of the time

5. A little of the time

6. Hardly any of the time

7. None of the time

**76. In the last 2 weeks, have you had a lot of energy?**

1. None of the time

2. Hardly any of the time

3. A little of the time

4. Some of the time

5. A good bit of the time

6. Most of the time

7. All of the time

**77. In the last 2 weeks, have you worried that your cough may indicate a serious illness?**

1. All of the time

2. Most of the time

3. A good bit of the time

4. Some of the time

5. A little of the time

6. Hardly any of the time

7. None of the time

**78. In the last 2 weeks, have you been concerned that other people think something is wrong with you,**

**because of your cough?**

1. All of the time

2. Most of the time

3. A good bit of the time

4. Some of the time

5. A little of the time

6. Hardly any of the time

7. None of the time

**79. In the last 2 weeks, my cough interrupted conversation or telephone calls.**

1. Every time

2. Most times

3. A good bit of the time

4. Some of the time

5. A little of the time

6. Hardly any of the time

7. None of the time

**80. In the last 2 weeks, I feel that my cough has annoyed my partner, family or friends.**

1. Every time I cough

2. Most times when I cough

3. Several times when I cough

4. Some times when I cough

5. Occasionally when I cough

6. Rarely

7. Never

**CONSENT TO USE MY DATA, WHICH HAS BEEN CATHERED IN MEDICAL REGISTERS**

You are able to participate in this questionnaire survey also without giving your personal identification data.

**Can my data in medical registers (Statistics Finland, the Cancer Registry, and the National Institute for Health and Welfare) be utilised in association with the present study?**

1 no. You can now submit the questionnaire without filling in the next sections – please click here___

2 yes. In that case, we ask you to fill in the next sections:

**Cough in Savo and Central Finland**

**Unit for Medicine and Clinical Research, Pulmonary Division, Kuopio University Hospital, Kuopio,**

I have been requested to participate in the above research project, which is intended to determine cough prevalence and to clarify factors that influence the prevalence and the consequences of cough in the population living in Eastern and Central Finland. I have read and understood the written information I have received about this research project. I have been given the opportunity to contact the researchers should I wish to receive further information. The information that I have received has given me a satisfactory understanding about this research project and about the way in which the data in the project will be gathered, handled and distributed. I was given a sufficient amount of time to consider whether or not I would participate in this project. I have been provided with sufficient information about my rights, the goals of this research project, how it will be implemented as well as the advantages and risks associated with this research project. I have not been coerced nor given any incentives to participate in this research project.

I understand that my participation is voluntary. I am aware that I can withdraw my permission to participate in this research project at any time and without giving any reason for my withdrawal. I am aware that my responses will be handled confidentially and not provided to any outside parties. I am aware that should I decide to interrupt or withdraw my permission to participate in this research project, any data which has been collected prior to my withdrawal/ refusal to continue can be included in the project’s research material.

Name of the participant _________________________________

Identity number of the participant________________________

Address of the participant___________________________________________________________

Telephone number of the paricipant______________________

Date________________________

You can now submit your responses by clicking here: ____
