# Supplementary material for: Cluster analysis in 975 patients with current cough identifies a phenotype with several cough triggers, many background disorders, and low quality of life
Source: Respir Res. 2020 Aug 20;21:219. doi: 10.1186/s12931-020-01485-y (PMC7441640; doi:10.1186/s12931-020-01485-y)
Supplement: Supplementary file 2 — Additional file 2. The number of NbClust criteria suggesting the best number of clusters in the total population, among subjects living in Jyväskylä, and among subjects living in Kuopio. The maximal number is 24 criteria. [file 12931_2020_1485_MOESM2_ESM.docx]

Additional file 2. The number of NbClust criteria suggesting the best number of clusters in the total population, among subjects living in Jyväskylä, and among subjects living in Kuopio. The maximal number is 24 criteria

| **Suggested best number of clusters** | **Total population, number of criteria suggesting** | **Jyväskylä, number of criteria suggesting** | **Kuopio, number of criteria suggesting** |
| --- | --- | --- | --- |
| 1 | 0 | 0 | 0 |
| 2 | 12 | 10 | 11 |
| 3 | 6 | 3 | 7 |
| 4 | 3 | 1 | 2 |
| 5 | 0 | 0 | 0 |
| 6 | 0 | 0 | 0 |
| 7 | 0 | 0 | 0 |
| 8 | 0 | 2 | 0 |
| 9 | 0 | 1 | 1 |
| 10 | 0 | 2 | 1 |
| 11 | 0 | 2 | 0 |
| 12 | 0 | 0 | 0 |
| 13 | 1 | 0 | 1 |
| 14 | 0 | 2 | 0 |
| 15 | 2 | 1 | 1 |
