# Supplementary material for: Cluster analysis in 975 patients with current cough identifies a phenotype with several cough triggers, many background disorders, and low quality of life
Source: Respir Res. 2020 Aug 20;21:219. doi: 10.1186/s12931-020-01485-y (PMC7441640; doi:10.1186/s12931-020-01485-y)
Supplement: Supplementary file 3 — Additional file 3. The clusters and their defining variables among 520 subjects with current cough living in the town Jyväskylä. The ten most important variables are expressed, in order of importance. The order was defined by the p value obtained by Mann-Whitney U test or chi-square test between the clusters. The values are expressed by either means (standard deviations) or percentages, unless stated otherwise. [file 12931_2020_1485_MOESM3_ESM.docx]

Additional file 3. The clusters and their defining variables among 520 subjects with current cough living in the town Jyväskylä. The ten most important variables are expressed, in order of importance. The order was defined by the p value obtained by Mann-Whitney U test or chi-square test between the clusters. The values are expressed by either means (standard deviations) or percentages, unless stated otherwise.

| **Order** | **Variable** | **Cluster A**  **N = 323** | **Cluster B**  **N = 197** | **P value** |
| --- | --- | --- | --- | --- |
| 1 | Trigger sum | 2.66 (2.30) | 7.13 (2.36) | 1.54 e-51 |
| 2 | Number of cough background disorders | 0.27 (0.49) | 1.29 (0.75) | 4.05 e-48 |
| 3 | Idiopathic cough | 75.2 % | 12.7 % | 5.22 e-42 |
| 4 | LCQ physical domain | 5.32 (0.80) | 4.21 (0.83) | 6.79 e-38 |
| 5 | LCQ question 9 ^1^ | 6.04 (1.08) | 4.35 (1.52) | 3.42 e-34 |
| 6 | LCQ total score | 16.34 (2.51) | 13.14 (2.71) | 4.54 e-34 |
| 7 | Exhaust fumes as a cough trigger | 14.9 % | 62.9 % | 1.51 e-28 |
| 8 | Strong paints or fumes as a cough trigger | 18.3 % | 68.0 % | 2.57 e-28 |
| 9 | Poor indoor air quality as a cough trigger | 38.4 % | 88.8 % | 4.70 e-28 |
| 10 | Dyspnea with wheezing | 16.1 % | 65.0 % | 5.18 e-28 |

^1^ Leicester Cough Questionnaire question number 9: “In the last 2 weeks, exposure to paints or fumes has made me cough” with a 7-step scale from 1 = all of the time to 7 = none of the time.
