# Supplementary material for: Cluster analysis in 975 patients with current cough identifies a phenotype with several cough triggers, many background disorders, and low quality of life
Source: Respir Res. 2020 Aug 20;21:219. doi: 10.1186/s12931-020-01485-y (PMC7441640; doi:10.1186/s12931-020-01485-y)
Supplement: Supplementary file 5 — Additional file 5. The cluster analysis by excluding those background variables with no plausible biological association with cough among 975 subjects with current cough. The ten most important variables are expressed, in order of importance. The order was defined by the p value obtained by Mann-Whitney U test or chi-square test between the clusters. The values are expressed by either means (standard deviations) or percentages, unless stated otherwise stated. [file 12931_2020_1485_MOESM5_ESM.docx]

Additional file 5. The cluster analysis by excluding those background variables with no plausible biological association with cough among 975 subjects with current cough. The ten most important variables are expressed, in order of importance. The order was defined by the p value obtained by Mann-Whitney U test or chi-square test between the clusters. The values are expressed by either means (standard deviations) or percentages, unless stated otherwise stated.

| **Order** | **Variable** | **Cluster A**  **N = 609** | **Cluster B**  **N = 366** | **P value** |
| --- | --- | --- | --- | --- |
| 1 | Trigger sum | 2.64 (2.20) | 6.97 (2.32) | 4.19 e-98 |
| 2 | Number of cough background disorders | 0.31 (0.54) | 1.25 (0.75) | 2.72 e-79 |
| 3 | LCQ physical domain | 5.34 (0.74) | 4.22 (0.84) | 7.03 e-72 |
| 4 | Idiopathic cough | 72.7 % | 13.7 % | 1.83 e-70 |
| 5 | LCQ question 9 ^1^ | 6.07 (1.04) | 4.31 (1.52) | 9.54 e-68 |
| 6 | LCQ total score | 16.5 (2.38) | 13.1 (2.69) | 3.21 e-67 |
| 7 | LCQ psychological domain | 5.44 (0.93) | 4.29 (1.04) | 2.28 e-54 |
| 8 | Dyspnea with wheezing | 15.8 % | 64.5 % | 5.19 e-54 |
| 9 | Poor indoor air quality as a cough trigger | 38.6 % | 89.3 % | 6.06 e-54 |
| 10 | LCQ social domain | 5.72 (0.99) | 4.57 (1.10) | 10.0 e-51 |

^1^ Leicester Cough Questionnaire question number 9: “In the last 2 weeks, exposure to paints or fumes has made me cough” with a 7-step scale from 1 = all of the time to 7 = none of the time.
